# Optimising frontline learning and engagement between consultant-led neonatal teams in the West Midlands: a survey on the utility of an augmented simulation training technique

**DOI:** 10.1186/s41077-021-00181-1

**Published:** 2021-08-28

**Authors:** Thillagavathie Pillay, Lynsey Clarke, Lee Abbott, Pinki Surana, Asha Shenvi, Sanjeev Deshpande, Joanne Cookson, Matthew Nash, Joe Fawke, Vishna Rasiah, Jonathan Cusack

**Affiliations:** 1 University Hospitals of Leicester NHS Trust, Leicester, UK; 2 Faculty of Science and Engineering, RIHS, University of Wolverhampton, Wolverhampton, UK; 3 College of Life Sciences, University of Leicester, Leicester, UK; 4 West Midlands Neonatal Operational Delivery Network, Solihull, UK; 5 University Hospitals of North Midlands NHS Trust, Stoke-on-Trent, UK; 6 Faculty of Medicine and Health Sciences, University of Keele, Keele, UK; 7 University Hospitals Birmingham NHS Foundation Trust, Birmingham, UK; 8 Shrewsbury and Telford Hospital NHS Trust, Shrewsbury, UK; 9 Birmingham Women’s and Children’s NHS Foundation Trust, Birmingham, UK

**Keywords:** Neonatology, Networks, Neonatal networking, Neonatal unit designation, Consultant training

## Abstract

**Background:**

In England, neonatal care is delivered in operational delivery networks, comprising a combination of the Neonatal Intensive Care (NICU), Local-Neonatal (LNU) or Special-Care Units (SCU), based on their ability to care for babies with different degrees of illness or prematurity. With the development of network care pathways, the most premature and sickest are mostly triaged for delivery in services linked to NICU**.** This has created anxiety for teams in LNU and SCU. Less exposure to sicker babies has resulted in limited opportunities to maintain expertise for when these babies unexpectedly deliver at their centre and thereafter require transfer for care, to NICU. Simultaneously, LNU and SCU teams develop skills in the care of the less ill and premature baby which would also be of benefit to NICU teams. A need for mutual learning through inter-unit multidirectional collaborative learning and engagement (hereafter also called neonatal networking) between teams of different designations emerged. Here, neonatal networking is defined as collaboration, shared clinical learning and developing an understanding of local systems strengths and challenges between units of different and similar designations. We describe the responses to the development of a clinical and systems focussed platform for this engagement between different teams within our neonatal ODN.

**Method:**

An interactive 1-day programme was developed in the West Midlands, focussing on a non-hierarchical, equal partnership between neonatal teams from different unit designations. It utilised simulation around clinical scenarios, with a slant towards consultant engagement. Four groups rotating through four clinical simulation scenarios were developed. Each group participated in a clinical simulation scenario, led by a consultant and supported by nurses and doctors in training together with facilitators, with a further ~two consultants, as observers within the group. All were considered learners. Consultant candidates took turns to be participants and observers in the simulation scenarios so that at the end of the day all had led a scenario. Each simulation-clinical debrief session was lengthened by a further ~ 20 min, during which freestyle discussion with all learners occurred. This was to promote further bonding, through multidirectional sharing, and with a systems focus on understanding the strengths and challenges of practices in different units. A consultant focus was adopted to promote a long-term engagement between units around shared care. There were four time points for this neonatal networking during the course of the day. Qualitative assessment and a Likert scale were used to assess this initiative over 4 years.

**Results:**

One hundred fifty-five individuals involved in frontline neonatal care participated. Seventy-seven were consultants, supported by neonatal trainees, staff grade doctors, clinical fellows, advanced neonatal nurse practitioners and nurses in training. All were invited to participate in the survey. The survey response rate was 80.6%. Seventy-nine percent felt that this learning strategy was highly relevant; 96% agreed that for consultants this was appropriate adult learning. Ninety-eight percent agreed that consultant training encompassed more than bedside clinical management, including forging communication links between teams. Thematic responses suggested that this was a highly useful method for multi-directional learning around shared care between neonatal units.

**Conclusion:**

Simulation, enhanced with systems focussed debrief, appeared to be an acceptable method of promoting multidirectional learning within neonatal teams of differing designations within the WMNODN.

**Supplementary Information:**

The online version contains supplementary material available at 10.1186/s41077-021-00181-1.

## Background

In England, neonatal care is delivered within Neonatal Operational Delivery Networks (ODNs [1, 2]). Within these ODNs, preterm and sick babies (neonates) are cared for in neonatal units of three designations: neonatal intensive care units (NICU), local neonatal units (LNU) and special care units (SCU). NICU have resources to cater for all babies, including the sickest, smallest and those born most prematurely [3, 4]; LNU, for babies who are usually >27weeks of gestation and not critically unwell; and SCU, for babies usually >32 weeks of gestation at birth, who require some medical intervention before being discharged home [3, 5]. Care provided in these units is consultant-led. In NICU, these consultants are neonatologists, whereas in LNU and SCU, they are general paediatricians [3], with an interest in neonatology, providing neonatal ward round *and* after-hours neonatal support in some, and *only* after-hours or emergency care neonatal support in others.

Since the emergence of ODNs and pathways diverting care for the very ill and premature baby into NICU [4], anxieties around potential ‘deskilling’ in LNU and SCU consultant-led teams have heightened (unpublished, anecdotal information, based on authors’ communications within the network). These network pathways [2–6] meant less exposure to the very preterm and sick baby for LNU and SCU teams and therefore limited opportunities to maintain skills for these when they do unexpectedly present to SCU and some LNU. An example of this variation in exposure to sick and preterm babies can be seen in our region in the West Midlands, where exposure to having had a preterm baby born <30 weeks of gestation, ranged from 1 case per year in one of our four SCU, to around 80 cases per year in one of our 5 NICU and 5 LNU (West Midlands Academic Health Science Network [7]).

Our senior non-NICU consultants expressed anxiety over this emergent trend (unpublished data). We reflected that while ODN structures [2–6], cot capacity and maternal choice of booking hospital reduced their experience of dealing with sick or extremely preterm babies, it did not prevent unexpected exposure to them, due to the unpredictability of preterm birth. We acknowledged that the logistics or providing intensive care was likely to be harder in a location that delivered it infrequently and for shorter periods of time. We considered it critical to ensure that the care provided in LNU and SCU remains optimal, while at the same time enabling NICU teams to support teams looking after sick babies outside of NICU.

For some teams, when dealing with a sick baby requiring shared care, the only engagement our consultants and teams from different designations had with each other was around the time of consultation in relation to the case. With the exception of this telephonic engagement a NICU at the time of need, there was a limited support available to SCU and some LNU teams supporting the individual sick baby. This meant that both our NICU and LNU/SCU teams had limited understanding of the perspectives of each other’s care and unit pressures, which is essential in making informed decisions regarding shared care for sick babies in our region.

At the same time, our senior colleagues in NICU were acknowledging that LNU and SCU teams, who were now consolidating their skills in managing slightly older, less critically ill babies [5], had a wealth of experience that could be tapped into by NICU teams in planning the shared care.

Providing clinical updates but at the same time understanding the variations in practice and systems issues (strengths and challenges) around caring for a critically ill baby in different locations was considered a useful way of bridging the knowledge and confidence gap between units. A 1-day training programme was developed in 2015 to explore this between teams. We hoped to design a programme that would improve relationships and community within the ODN. We focussed on creating a space that would allow for multidirectional learning, co-construction of organisational knowledge, open communication, increased understanding of peoples’ roles and the promotion of mutual respect for the diversity of skills that participants brought to the table. This approach is rooted in social constructivism [8] and well supported by organisational theories such as relational coordination [9]. The focus was on the utility of group activity through which meaningful learning would occur, where the learners were active participants and where the responsibility of knowledge acquisition/sharing lay across all members of the group. Here, we hoped for mutual co-construction of knowledge amongst all the learners.

Simulation around common clinical critical care cases was the vehicle used for this for shared learning. We utilised an extended systems focus during debriefing to consolidate professional multidirectional learning.

While no similar course existed in neonatology in England at the time exploring a systems focus promoting the relational aspect of care in a collaborative manner within networks, a similar model described in trauma care [10, 11] encouragingly supports the utility of this approach in our discipline.

We report on the user experience with this technique of supporting neonatal inter-team learning in the West Midlands.

## Methods

### The initiative

A faculty of facilitators, comprising consultants from NICU and LNU, advanced nurse practitioners and nurse practice educators from the Staffordshire Shropshire and Black Country Newborn and Maternity Network (SSBCNMN) and Southern West Midlands Maternity and Newborn Network (SWMNN), now called the West Midlands Neonatal Operational Delivery Network (WMNODN), was established in 2015. We developed a 1-day interactive course/programme called ‘Supporting the Sick Neonate (SSN)’, based on simulation sessions as described below. The sessions focussed on consultant-led decision support and their management of teams in clinical situations, followed by exploring systems issues through mutual discussions on the experiences in their units, limitations, difficulties, strengths and weaknesses, with shared care.

### Objective

Its overarching objective was to support consultant and team interaction, fostering shared learning, mutual respect and enhancing rapport between NICU, LNU and SCU teams. In this context, both facilitators and candidates were learning from each other.

### Peer review

Peer review support was sought at the outset from two additional NICU centres outside of the West Midlands. A mock run of the initiative was completed before the roll out of the programme. All Trusts were informed of the programme, and the neonatal networks encouraged consultants and team members involved in front-line neonatal care to participate in this as part of their essential continued professional development. Intense 1:1 discussion outlining the participatory, confidential and supportive nature of the sessions was held by the course director with consultant candidates in LNU and SCU, to allay anxieties of being under scrutiny during the day. All experiences within the scenarios were regarded as confidential.

### The sessions

The 1-day interactive course/sessions had four groups rotating through four clinical simulation scenarios (Table 1). Each group participated in a clinical simulation scenario, led by a consultant and supported by nurses and paediatric/neonatal doctors in training together with facilitators, with a further ~two consultants as observers within the group. All were considered learners. Consultant candidates took turns to be participants and observers in the simulation scenarios so that at the end of the day all had led a scenario.

### The systems focus following clinical simulation

A PEARLS approach and systems–focus [12, 13] was used. Following the clinical scenario, focussed debrief around the case and information sharing in the form of directive feedback and/or teaching, a further ~20 min of free interactive discussion was promoted. This was to explore systems issues between teams through collaborative sharing of experiences and practices across units and teams. This was inter-unit, collaborative multidirectional learning and engagement (hereafter called ‘neonatal networking’ as this was conducted within the Operational Delivery Neonatal Network’) and included observers who had not participated in the simulation, but formed part of the group. Variations in practice, limitations, challenges and systems limitations in dealing with similar complex babies in different designations of units in the West Midlands were variably discussed in this context. The overarching purpose of this day was to enable mutual understanding of clinical practice and systems issues, so that situationally, better decisions at the time of consultation could be made, for the benefit of the baby requiring care across multiple sites and for individual team/unit development. It was intended to build relationships and foster ongoing mutual respect for the variation in care provided, between LNU/NICU/SCU team members. A consultant focus was adopted to promote long-term engagement between units around shared care. There were four time points for neonatal inter-unit multi-directional collaborative engagement and learning, during the course of the day.

None of the sessions were graded. This was deliberate, to ensure that the participating consultants, especially senior LNU and SCU colleagues were not threatened by a feeling of being assessed.

### Participants

Facilitators were NICU or LNU consultants supported by an Advanced Neonatal Nurse practitioner, Clinical fellow and/or Nurse Educator.

#### Candidates

Each group comprised (i) 2–3 regional consultants from NICU, LNU and SCU and (ii) regional tier 1 and 2 trainees/advanced neonatal nurse practitioners/practice educator nurses. The latter made up the team around the consultant.

In the first 2 years of the programme, the junior members who were included to form this team were confederate faculty members—staff grade doctors, clinical fellows and advanced nurse practitioners working on tier 1 and tier 2 paediatric/neonatal rota within the West Midlands. In subsequent years, a small group of trainees rotating through neonatology at the time were recruited as candidates. This change was effected following consideration that having a confederate junior team who were therefore very familiar with the scenarios would put undue pressure on the consultant candidates. It was felt that a team that was naïve to the clinical scenario at simulation would perform in a manner that better represents realities on the ground and that this was more likely to yield areas for learning and reflection from the course. In order to support neonatal nurses undertaking a Qualified in Speciality (QIS) course with Keele University, they were included to participate in the role of the neonatal nurse/observer in the scenarios in 2017 and 2018.

Within each session, one of the three consultant candidates led each clinical scenario. The ‘team around the consultant’ participated in every scenario. In each session, the remaining two consultant candidates not leading the scenario stood back as observers for the scenario and case-focussed debrief. However, all, including the facilitators, the team around the consultant and the observers contributed actively to systems focussed discussions that followed. This was multi-directional engagement, collaboration and learning.

The four sessions were run in parallel, in four separate clinical skills areas within the West Midlands Learning Centre. Each session lasted approximately 90 min.

### The evaluation

Participants were asked to complete a survey form, specifically designed for the course (see supplementary file), anonymously at the end of their 1-day programme, from the courses in October 2015, June 2016, January 2017 and Sept 2018. The survey was created to assess whether the format for training was relevant to consultants and teams around consultants, whether there was shared learning and to pick up ideas for improvement driven by the learners. The first year served as the pilot, after which, it continued. The Likert scale was used to assess acceptability, and the free text boxes intended to identify areas around which we could develop the training further. As this was an unfunded project, we did not formally validate the survey questions.

Graded responses using a Likert scale along the following themes were manually extracted around:

- Whether this met their educational needs and the appropriateness of the techniques in the sessions as part of their learning
- Their opinion on centre-based vs point of care neonatal training
- Value of inter-unit multi-directional collaborative engagement and learning with colleagues from the region through these modified simulation sessions

Free text responses were manually reviewed and themes explored where whether this (a) met the educational needs and the appropriateness of the learning, (b) whether the learners appreciated the multi-directional learning promoted, (c) met their expectations for the future and (d) recommendations sought.

## Results

A total of 155 individuals participated in this West Midlands initiative over the 4-year period. These participants included facilitators and candidates together. Seventy-seven were consultants involved in newborn clinical care, (57 as candidates and 20 as facilitators), 47 were neonatal/paediatric team members and 31 were neonatal nurses in training. The distribution of participants is detailed in Table 2.

**Table 1:** Example scenarios and workshops offered in the supporting the sick neonate course

**Example sessions**	*Themes identified as requiring support (SSN 2015-2018)* • A moderately preterm baby in a LNU develops increased work of breathing and an increasing oxygen requirement on non-invasive ventilator support. • A term baby with meconium aspiration is hypoxic. • Dealing with failed resuscitation in delivery suite: support for the mother, family, team and defining local processes. A term baby with a difficult airway—setting up a difficult airway trolley in your local unit. *Themes identified for the future:* • A moderately preterm baby with respiratory distress due to presumed surfactant deficiency—less invasive surfactant replacement. • Managing a subgaleal bleed • Sudden unexpected postnatal collapse • The blue baby • Dealing with the extremely premature baby born outside of a maternity service attached to a NICU
**Workshops**	• Interosseous access for intravenous fluids • Videolarygoscopy in term and preterm baby • Seldinger technique chest drain insertion • Ultrasound-guided peripheral long line insertion 2018 session only) • Laryngeal mask airway insertion • The difficult airway (as part of a scenario-based workshop)

**Table 2:** Distribution of neonatal team learners on SSN course 2015–2018

	Faculty	Candidates	Support
**Consultants**			
NICU Consultant Neonatologist	12	5	
LNU Consultant Paediatrician providing neonatal and paediatric care	2	29	1
LNU Consultant Paediatrician providing only neonatal care (split rota)	4	6	1
SCU Consultant Paediatrician providing neonatal and paediatric care	0	14	0
**Total**	**18**	**52**	**2**
**Other neonatal team members**			
Practice educators	3	0	1
Tier 1 and tier 2 trainees	1	18	0
ANNP/staff grade doctors	3	21	0
Neonatal nurses in training	0	31	0
**Total**	**7**	**70**	**1**

*SSN* supporting the sick neonate, *LNU* local neonatal unit, *SCU* special care unit, *ANNP* advanced neonatal nurse practitioners, *PEM* Paediatric Emergency Care Medical Consultants, who see neonatal emergencies as part of their practice

**Table 3:** Graded responses to the supporting the sick neonate course (1 = strongly disagree, 2=disagree, 3= no opinion, 4 = agree, 5= strongly agree)

Question	Grade of response (%)					Total (***n***=)	Median grade of response
For consultants this is an appropriate way of engaging in adult learning	0	1	3	32	64	81	5
Getting together nurses, trainees and consultants to learn together in a team is useful	1	0	1	21	77	97	5
I do not mind not knowing all the members of my tier 1/2 simulation team, as this may be the case in reality in my teams at times of doctors’ changeovers	3	3	4	35	54	96	5
I would prefer only to undertake simulation at my base hospital, and don’t mind that I do not get to engage with other consultants from other hospitals	31	19	16	17	17	94	2
I would be happy to attend around 2 meetings a year in course-based adult learning, such as this	13	15	7	34	31	93	4
Consultant training should not only be about clinical management at the bedside, but networking and forging communication links between teams in newborn networks	2	3	4	39	51	90	5

**Table 4:** A selection of participant responses to the SSN networking model: a focus on appropriateness of method, utility of networking time and expectations for the future

Themes	Participant quotes
**General**	‘Fabulous day’; ‘excellent course’; ‘will be back for more’; ‘well executed’, ‘learnt so much through the day both clinically and human factors’, ‘brilliant’
**Meeting educational need and appropriateness of learning method**	‘Essential practice update for SCU/LNU consultants; appropriately pitched to accentuate learning’ ‘topics which were very relevant to me working in the level I work at’ ‘Very good quality of education , practical scenarios, not patronising, well led’ **‘**All learning objectives met in a good learning environment’ ‘Very good value’, ‘very good content’, ‘very useful and practical’ ‘All speakers … had extensive skills and experience which they were able to share’
**Multi-directional learning and networking support**	‘Brilliant and non-threatening’; ‘Our ideas were listened to’ ‘Feedback and reflection time very useful’ ‘Appreciated the 20 minute time for reflection and communication.’ ‘Good update learning from others’ ‘Networking friendly and non- threatening’ ‘Excellent way to learn and will help us to do our own simulations at our hospital’ ‘Interesting to see how different consultants react in different situations’. ‘Appreciated ‘different doctors from different units getting together’, ‘discussions with rest of the group’ ‘Feedback support was very good, non-judgmental’ ‘Some useful sharing of messages’ ‘Very useful ideas I have taken back for local in house simulation sessions’ ‘Good refresher for LNU as I have limited exposure’ ‘Thought provoking, solved some of my queries’ ‘The information shared highlighted our need to find out what our own local policies are.’ ‘It was good being with nurses, doctors and ANNPS’, “appreciated the communication, and networking with friends and colleagues’, and ‘meeting old friends’,
**Expectations for the future and recommended improvements**	‘This highlights the divide between LNU/NICU, and we need more NICU’ participants as ‘candidates’ ‘More opportunities for candidate to share experiences with remainder of team is needed’ ‘I think it would be useful to have more juniors and nurses to be able to change the team more between scenarios’ ‘Include more scenarios’ ‘I do not feel there is any improvement to be made’ ‘None. Superb course’

Four of the five courses were evaluated, and 100 participants (80.6%) of 124 eligible in the four courses, completed the survey anonymously. All felt that the learning objectives of the course were met. Seventy-nine percent felt that the course was highly relevant, a further 16% that it was mostly relevant and 5% that it was fairly relevant. The graded responses on perspectives of the course are detailed in Table 3. Ninety-six percent felt that for consultants, this was an appropriate way of engaging in adult learning. Ninety-eight percent agreed that consultant training encompassed more than just clinical management at the bedside but included forging communication links between teams in newborn networks. Eighty-nine percent did not mind not knowing all the members of tier 1 and tier 2 medical teams as this may represent the case in reality at the time of doctors’ changeover in rotation. Fifty percent of those who responded did not agree with simulation being done at the base hospital only, and on not engaging with other consultants from other hospitals; 16% had no opinion on this.

A review of the free-text responses revealed candidates feeling that their ‘ideas were listened to’, that it was ‘good being with nurses, doctors and ANNPS’, that they ‘appreciated the communication, and networking with friends and colleagues’ and ‘meeting old friends’, and that the ‘networking was friendly and ‘discussions with rest of the group were non-threatening’. They felt ‘supported’ that participants had ‘incredible knowledge, extensive skills and experience which they were able to share’; that this was a ‘good update’; and that they appreciated ‘learning from others’. The feedback given during the sessions were considered ‘non-judgmental’ and ‘the information shared highlighted their need to find out what their own local policies were.’ They ‘appreciated the 20-min time for reflection and communication.’ Table 4 displays some common themes around the educational meeting, the inter-unit multi-directional collaborative engagement and learning (neonatal networking), and expectations and recommendations for the future from the participants.

## Discussion

We describe our early experience with an initiative within the neonatal ODN in the West Midlands, using simulation with an extended freestyle systems-focus discussion as part of the debrief. It centred on shared learning between units against the background of critical clinical scenarios faced by the care provided at LNU/SCU and NICU. The initiative attempted to firstly address concerns around potential deskilling of consultants and limited collaborative engagement between teams caring for sick newborn babies in our region, outside of the NICU environment. Secondly, it attempted to promote multi-directional learning in a safe, non-threatening environment, away from assessment between NICU, LNU and SCU teams, with a heavy focus on consultant-based learning. This learning was structured around understanding the strengths, challenges, experiences and perspectives of learners across NICU, LNU and SCU in a multidirectional manner and with mutual respect for each other in the care they provided.

Two theories ground this work. The first, a relational coordination theory [13] in which the ‘task’ at hand, is optimal care for the ill baby requiring shared care between neonatal units. The coordination of this task ideally occurs through a network of relational communications between teams involved in providing this care. To effect the outcome of the task (whether this is the baby’s clinical condition improving, or supporting re-orientation of care away from life-sustaining support), coordination, mutual respect, shared knowledge and shared goals are the priority. As the consistent feature in clinical care, consultants act as ‘nodes’ through which this occurs. In relational coordination model, formal organisational structures (here, the WMNODN) support the fundamentals of this model i.e. promotion of frequent, timely, accurate and problem-solving communications. This describes our initiative within the WMNODN. The second is social constructivism [9] in which learning is considered a social process, occurring through discussions between members of different teams and across different units. Instructional guidance, in this case, the simulation and focussed debrief, followed by shared discussions of experiences and practices promotes the acquisition of shared knowledge as the group interacts in the learning process.

This initiative used a central venue as its base. The core concept being non-hierarchical engagement between teams for the benefit of neonatal care where the WMNODN represented the ‘team’ and that its consultants and juniors and support its staff and its members. The combination of LNU, SCU and NICU consultants in the same learning environment was not simply to offer advanced resuscitation training [4], but to focus on common principles of care and to promote interunit collaborative engagement and communication through a mutual understanding and respect for varying exposures to sick babies at different units, their practices, stressors and challenges, and the need to support each other.

A heavily weighted consultant focus was considered the best way to enable this. Consultants represent the least-mobile component of the clinical workforce overall, around which the ‘teams’ could be built. We wanted to promote an environment where the voice on the other end of the telephone during late-night transfers/shared care consultant/senior staff discussions was familiar, and the decisions made in the best interest of the critically ill baby, better appreciated by both teams.

The added time facilitated further discussion that broadened out to include local and ODN issues. It is here that we believe the greatest strength of the sessions lay. It allowed team members of differing designations to relate their experiences, reflections, learning and questions and appeared to have been received and adopted positively. Learning around a common theme in a simulation session proved a successful adjunct to promoting engagement between consultants from different designations within the region.

Given that these simulations dealt with unstable babies that potentially need a transfer, one could consider that there are 3 potential ‘point-of-care’ locations—the referring unit, transport team and receiving unit. It would be very difficult to conduct a true ‘point of care simulation’ encompassing all 3 locations. In our programme, the choice of location was driven by the learning and engagement we set out to achieve. More evidence that functional alignment, as opposed to the location of simulation is relevant in learning [14], argues our case for not having a point of care simulation for this team-building exercise. It is reassuring from Tables 3 and 4 to see no major disagreement with the lack of point of care training in this course.

A limitation of this project was that it involved a large faculty and was intense in its requirement for time, coordination and resources to deliver. This may impact on its sustainability over time. Future projects in our region must assess the benefits to patient care through analysis of the impact of this form of intensive training on knowledge, skills, attitude and behaviours of staff around unit practices and shared care (discussions, triage and transfers), transport metrics and patient outcomes. Also, while we had LNU and NICU consultants as facilitators for this initiative, we acknowledge that future initiatives should incorporate the strengths of SCU in facilitating sessions in areas of strength over those of a NICU/LNU.

At the time of development, we considered alternative strategies such as a point of care training, and using a mobile outreach team, but discarded this as more labour intensive, and less likely to enable the same neonatal networking/engagement as with working with other colleagues from different LNU, SCU and NICU teams. The ‘cross-fertilisation of ideas’ including the comradery and vigour for change that accompanies a realisation that other units are experiencing similar difficulties and successes were considered more appealing using a central base for the course. Thematic responses from Table 4 suggest that this is the case; however, we have not studied any other NICU, LNU and SCU consultant-focussed strategies for our region yet, to be sure which will be most sustainable and most translatable improving quality of neonatal care going forward. This is an area for future research. The potential that point-of-care training offers, in engaging the team including neonatal nurses, is acknowledged and will be a direction pursued, for the future. Other less costly initiatives that do not include simulation should also be considered as options for study.

Technical and non-technical skill training is important to maintain for all designations of neonatal unit care in the region [15, 16]; how best to achieve this, while understanding local systems issues, and promoting learning within an unthreatening supportive environment for all, including consultants, is the challenge going forward. Our perception is that this will best be maintained through a focus on social constructivism and relational coordination, around which learning objectives for neonatal teams can be met.

## Conclusion

For the West Midlands, simulation, enhanced with systems focussed debrief, provided a useful platform for neonatal inter-unit multidirectional collaborative engagement (‘neonatal networking’), promoting shared learning between LNU, SCU and NICU consultants and neonatal teams. Future challenges will be how best to use the principles of SSN in providing multi-directional support for the babies who are acutely ill in non-specialist settings outside of a NICU, in a format that is sustainable, and widens the learner group while preserving the focus on the consultant learner. Research around the impacts of such programmes, must include more rigorous qualitative study, including methods to streamline resources required to deliver such a programme.

## Supplementary Information

**Additional file 1.**

## Data Availability

All data generated or analysed during this study are included in this published article. The Supplementary File contains the survey questionnaire.

## Abbreviations

LNU: Local neonatal unit
SCU: Special care unit
NICU: Neonatal intensive care unit
ODN: Operational Delivery Network
SSN: Supporting the sick neonate
SSBCNMN: Staffordshire Shropshire and Black Country Newborn and Maternity Network
SWMNN: Southern West Midlands Maternity and Newborn Network
WMNODN: West Midlands Neonatal Operational Delivery Network

## References

[1] NHS England. Operational delivery networks. https://www.england.nhs.uk/ourwork/part-rel/odn/ Accessed 2 Jul 2021

[2] NHS England. NHS Commissioning. Specialised services; E08 Neonatal Critical Care. https://www.england.nhs.uk/commissioning/wp-content/uploads/sites/12/2015/01/e08-serv-spec-neonatal-critical.pdf Accessed 2 Jul 2021

[3] Service Standards for Hospitals Providing Neonatal Care (3rd edition) (2010)|British Association of Perinatal Medicine (bapm.org) https://www.bapm.org/resources/32-service-standards-for-hospitals-providing-neonatal-care-3rd-edition-2010 Accessed 2 Jul 2021

[4] Optimal Arrangements for Neonatal Intensive Care Units in the UK (2014)|British Association of Perinatal Medicine (bapm.org) https://www.bapm.org/resources/31-optimal-arrangements-for-neonatal-intensive-care-units-in-the-uk-2014 Accessed 2 Jul 2021

[5] Optimal arrangements for local neonatal units and special care units in the UK (2018)|British Association of Perinatal Medicine (bapm.org) https://www.bapm.org/resources/2-optimal-arrangements-for-local-neonatal-units-and-special-care-units-in-the-uk-2018 Accessed 2 Jul 2021

[6] Implementing the recommendations of the neonatal critical care transformation review. NHS England. https://www.england.nhs.uk/wp-content/uploads/2019/12/Implementing-the-Recommendations-of-the-Neonatal-Critical-Care-Transformation-Review-FINAL.pdf Accessed 2 Jul 2021

[7] Gillies J, Hodgetts Morton VA, Jasim S, Fox C, Broggio P, Pillay T (2021). Effecting a national implementation project through distributed leadership in the West Midlands: rising to the spread challenge. BMJ Open Qual.

[8] Akpan VI, Igwe UA, Mpamah IBI, Okor CO (2020). Social constructivism: implications on teaching and learning. Br J Educ.

[9] Gittel JH. Relational coordination. Org Behav. 2015;11. 10.1002/9781118785317.weom110025.

[10] Brazil V, Purdy E, Alexander C, Matulich J (2019). Improving the relational aspects of trauma care through translational simulation. Adv Simul.

[11] Nickson CP, Petrosoniak A, Barwick S, Brazil V (2021). Translational simulation: from description to action. Adv Simul.

[12] Eppich W, Cheng A (2015). Promoting Excellence and Reflective Learning in Simulation (PEARLS): development and rationale for a blended approach to health care simulation debriefing. Simul Healthc.

[13] Dubé MM, Reid J, Kaba A, Cheng A, Eppich W, Grant V, Stone K (2019). PEARLS for systems integration: a modified PEARLS framework for debriefing systems-focused simulations. Simul Healthc.

[14] Brazil V (2017). Translational simulation: not ‘where?’ but ‘why?’ A functional view of in situ simulation. Adv Simul.

[15] Brown JM, Mitchell TK, Kirkcaldy AJ, Shaw BNJ (2017). Assessing the impact of the advanced resuscitation of the newborn infant course. Infant.

[16] RCUK (2021). Advanced resuscitation of the newborn infant.

