# Supplementary material for: Optimising frontline learning and engagement between consultant-led neonatal teams in the West Midlands: a survey on the utility of an augmented simulation training technique
Source: Adv Simul (Lond). 2021 Aug 28;6:29. doi: 10.1186/s41077-021-00181-1 (PMC8401370; doi:10.1186/s41077-021-00181-1)
Supplement: Supplementary file 1 — Additional file 1. [file 41077_2021_181_MOESM1_ESM.docx]

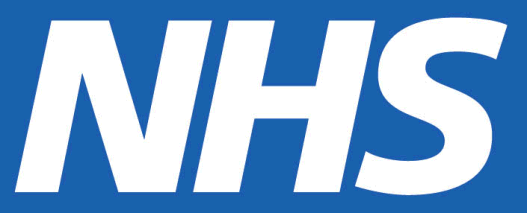


Staffordshire, Shropshire & Black Country

Newborn and Maternity Network


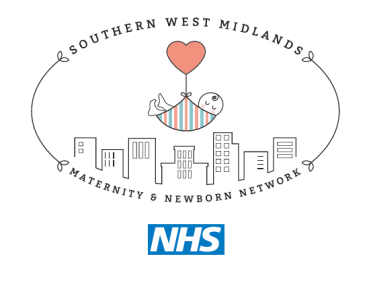


**Evaluation Form**

| **Event** | **Supporting the Sick Neonate Course** |
| --- | --- |
| **Venue** | **Walsall Manor Learning and Conference Centre,**  **Walsall Manor Hospital** |
| **Date** |  |
| **Name (Optional)** | **Consultant Candidate/Team member/Facilitator** |

**Were the printed learning aims/objectives of the course met? Yes No**

**Comments: (optional)**

**Was there any bias or conflict of interest in the course? Yes No**

**Comments: (optional)**

**Were the speakers/instructors knowledgeable? Yes No**

**Comments: (optional)**

**How do you rate the relevance of this CPD activity to your educational needs?**

**Circle one option**

| **1** | **2** | **3** | **4** |
| --- | --- | --- | --- |
| **Not Relevant** | **Fairly Relevant** | **Mostly of Relevance** | **Highly Relevant** |

**Comments: (optional)**

**How do you rate the overall quality of the education offered by this event?**

**Circle one option**

| **1** | **2** | **3** | **4** |
| --- | --- | --- | --- |
| **Not Relevant** | **Fairly Relevant** | **Mostly of Relevance** | **Highly Relevant** |

**Comments: (optional)**

**Please circle the most appropriate score for the following (4 being excellent and 1 being poor).**

| **Title of Session** | **Good Poor** | | | |
| --- | --- | --- | --- | --- |
| **Scenario 1** | **4** | **3** | **2** | **1** |

**Comments: (optional)**

| **Title of Session** | **Good Poor** | | | |
| --- | --- | --- | --- | --- |
| **Scenario 2: Simulation and Discussion** | **4** | **3** | **2** | **1** |

**Comments: (optional)**

| **Title of Session** | **Good Poor** | | | |
| --- | --- | --- | --- | --- |
| **Scenario 3: High Fidelity Simulation** | **4** | **3** | **2** | **1** |

**Comments: (optional)**

| **Title of Session** | **Good Poor** | | | |
| --- | --- | --- | --- | --- |
| **Difficult Airway Workshop** | **4** | **3** | **2** | **1** |

**Comments: (optional)**

| **Overall Co-ordination, Organisation and Future Direction** | **Good Poor** | | | |
| --- | --- | --- | --- | --- |
|  | **4** | **3** | **2** | **1** |

**Comments: (optional)**

**Did the Course meet its objective of bringing together teams?**

What aspects did you appreciate?

What could be improved upon?

**Rate the following 1-5 (1 = strongly disagree, 2=disagree, 3= no opinion, 4 = agree, strongly agree)**

1. For consultants this is an appropriate way of engaging in adult learning

| 1 | 2 | 3 | 4 | 5 |
| --- | --- | --- | --- | --- |

1. Getting together nurses, trainees and consultants to learn together in a team is useful.

| 1 | 2 | 3 | 4 | 5 |
| --- | --- | --- | --- | --- |

1. I do not mind not knowing all the members of my tier1/2 simulation team, as this may be the case in reality in my teams at times of doctors’ changeovers.

| 1 | 2 | 3 | 4 | 5 |
| --- | --- | --- | --- | --- |

1. I would prefer only to undertake simulation at my base hospital, and don’t mind that I do not get to engage with other consultants from other hospitals

| 1 | 2 | 3 | 4 | 5 |
| --- | --- | --- | --- | --- |

1. I would be happy to attend around 2 meetings a year in course-based adult learning, such as this

| 1 | 2 | 3 | 4 | 5 |
| --- | --- | --- | --- | --- |

1. Consultant training should not only be about clinical management at the bedside, but networking and forging communication links between teams in newborn networks

| 1 | 2 | 3 | 4 | 5 |
| --- | --- | --- | --- | --- |

**If this course appealed to you, please suggest topics for the next cycle of training**

**What improvements do you feel could be made for future events?**

**In this free text space, write down how/if you think this course will influence your**

1. Clinical practice
2. Situational awareness
3. Human factor awareness
4. Teaching junior members and colleagues on your team

Please indicate if you have any objections to the faculty approaching you in 2 months’ time for further electronic or telephonic feedback

**Thank you for taking the time to complete this evaluation form.**

**If you wish to discuss any issues relating to this event please contact**

**XXXXXX**
